# Causal association between inflammatory bowel disease and hidradenitis suppurativa: A two-sample bidirectional Mendelian randomization study

**DOI:** 10.3389/fimmu.2023.1071616

**Published:** 2023-01-26

**Authors:** Bingzhou Bao, Chao Zhu, Jian Shi, Canxing Lu

**Affiliations:** ^1^ Department of Anorectal, The First Affiliated Hospital of Anhui University of Chinese Medicine, Hefei, China; ^2^ College of Traditional Chinese Medicine, Anhui University of Chinese Medicine, Hefei, China

**Keywords:** inflammatory bowel disease, ulcerative colitis, Crohn’s disease, hidradenitis suppurativa, Mendelian randomization

## Abstract

**Background:**

Epidemiological studies have revealed a link between inflammatory bowel disease (IBD) and hidradenitis suppurativa (HS). To determine whether IBD and HS are causally related, we used the Mendelian randomization (MR) approach.

**Methods:**

A two-sample MR was performed using an analysis of 12,882 patients and 21,770 controls with IBD and its main subtypes, ulcerative colitis (UC) and Crohn’s disease (CD). A total of 409 cases and 211,139 controls without hidradenitis suppurativa (HS) were included in the data for this condition from various GWAS investigations. Odds ratios (ORs) with 95% confidence intervals (CIs) are used to estimate causal effects.

**Results:**

The study assessed the causal relationship between HS and IBD in both directions. The risk of HS was increased by IBD (IVW OR = 1.34, 95% CI = 1.20-1.49, *p* = 2.15E-07) and, in addition, HS was affected by UC (IVW OR = 1.27, 95% CI = 1.13-1.43, *p* = 8.97E-04) and CD (IVW OR = 1.18, 95% CI = 1.08-1.29, *p* = 4.15E-04). However, there was no evidence of a causal relationship between HS and IBD or its subtypes (IBD IVW OR = 1.00, 95% CI = 0.96-1.05, *p* = 0.85; UC IVW OR = 0.99, 95% CI = 0.95-1.03, *p* = 0.65; CD IVW OR = 1.03, 95% CI = 0.98- 1.07, *p* = 0.28).

**Conclusion:**

This study demonstrates that IBD and its subtypes have a causal effect on HS, whereas HS does not affect IBD. Gut-skin axis interactions may help to understand this association. Nevertheless, further studies are needed to clarify the pathophysiology of the causal relationship between IBD and HS.

## Introduction

Inflammatory bowel disease (IBD), also known as ulcerative colitis (UC) and Crohn’s disease (CD), is characterized by chronic inflammation of the gastrointestinal tract (1, 2). North American and European developed nations witnessed a steep rise in IBD in the latter half of the twentieth century (3). Symptoms of ulcerative colitis (UC) include rectal bleeding, diarrhea, abdominal pain, a fever, anemia, and weight loss (4, 5). In addition to diarrhea and abdominal pain, Crohn’s disease (CD) can affect any part of the digestive tract (6). Many patients will face chronic pain, depressive anxiety, and embarrassment due to fecal incontinence over time (7, 8). A growing number of patients worldwide suffer from Inflammatory bowel disease (IBD), especially in newly industrialized nations, and a severe and life-long burden is associated with it (9).

The skin condition known as hidradenitis suppurativa (HS) affects the hair follicle and is characterized by painful nodules, abscesses, sinus tracts, and scarring. Some evidence suggests that IBD and HS share a joint clinical presentation, genetic susceptibility, and immunological profile (10, 11). Clinically, both disorders are characterized by developing sinus tracts in the skin and gut, scarring, and aseptic abscesses in the perineum and inguinal area (12, 13). Moreover, both HS and IBD lead to an increased prevalence of spondyloarthropathies (14, 15). Case-control studies from the United States (16) and Finland (17) have demonstrated a potential relationship between hidradenitis suppurativa and IBD. Interestingly, previous studies have shown this association to exist only in Crohn’s disease, and ulcerative colitis does not appear to be associated with hidradenitis suppurativa (10, 18). However, the above studies are retrospective observational studies based on survey data collected for non-statistical reasons. Different measurement mistakes and potential biases (e.g., monitoring discrimination) might reverse the causality and make it difficult to distinguish the genuine causes from the effects.

Mendelian randomization (MR) can eliminate the confounding bias inherent in observational studies by using genetic variation as an index of causality. Different genotypes determine different intermediate phenotypes, and if this phenotype represents an exposure characteristic of an individual, the association effect with genotype and disease can describe the impact of exposure factors on illness, which is not influenced by confounding factors and reverse causal associations as in traditional epidemiological studies, since alleles follow the principle of random assignment (19, 20). We used a two-sample bidirectional MR analysis in the current study to determine the relationship between IBD (UC and CD) and HS.

## Materials and methods

**Figure 1:**
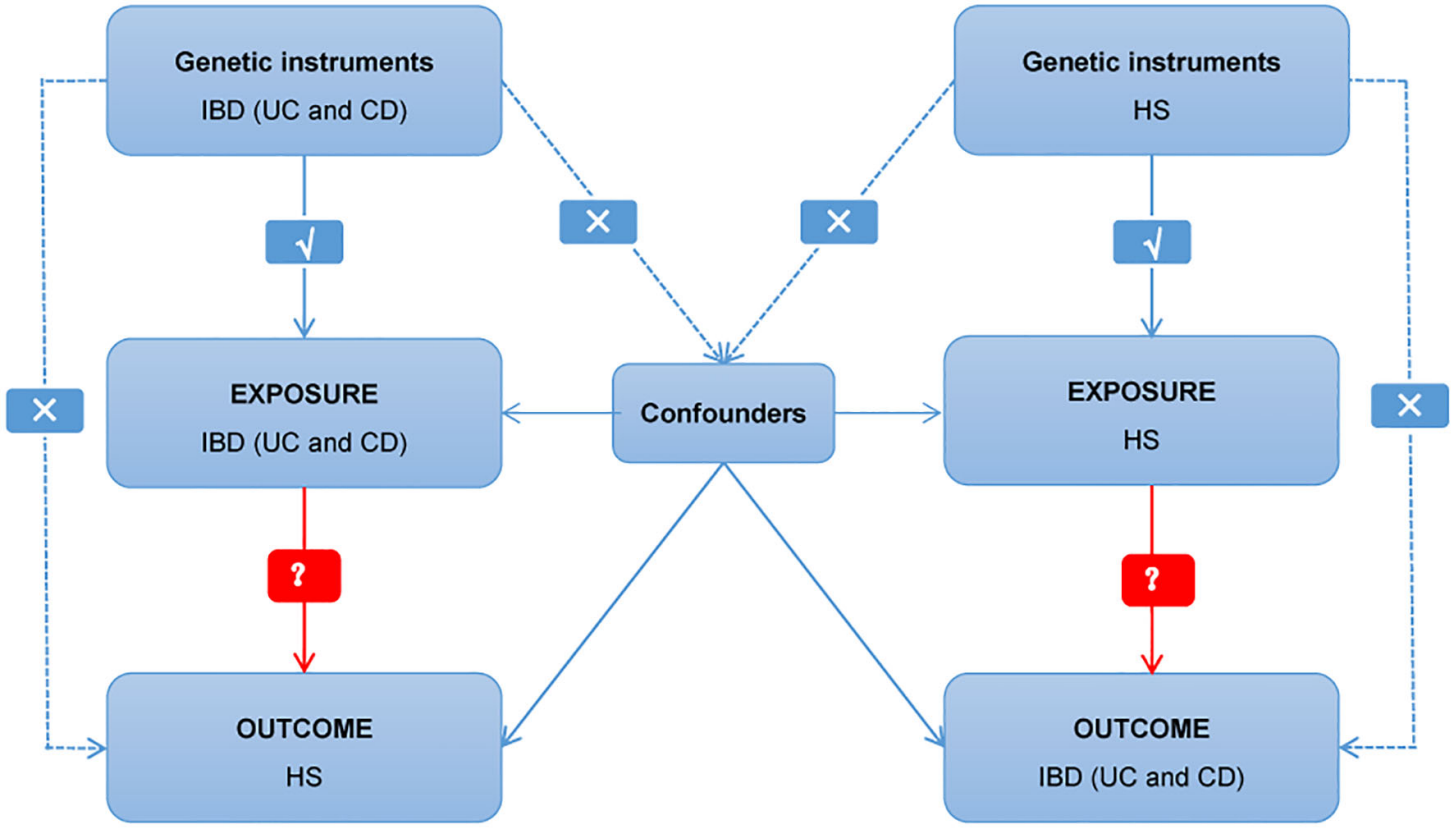
Diagram of critical assumptions for MR analysis. The solid line indicates that genetic instruments (SNPs) are related to exposure and can only affect the results through exposure. The dashed line indicates genetic instruments (SNPs) independent of any confounding variables between the results. IBD, inflammatory bowel disease; UC, ulcerative colitis; CD, Crohn’s disease; HS, hidradenitis suppurativa.

Multiple single nucleotide polymorphisms (SNPs) that represent genetic variation were used in a two-sample MR study. Three critical assumptions must be met (**Figure 1**): 1. Instrumental factors immediately associated with exposure; 2. Instrumental variables and other confounding factors are not relevant; 3. Genetic variation affects outcomes through exposure only (21). The bidirectional causal connection between HS and IBD (including UC and CD subtypes) was evaluated using MR analysis.

### Data sources

Databases generated by genome-wide association studies (GWAS), including IEU openGWAS, and GWAS Catalog, were searched, and eligible datasets were retrieved and exported. Since the data used are publicly available in the database, no additional ethical approval was needed in this case.

The IBD study involved 96,486 individuals, 89.8% of whom were of European ancestry. Only individuals of European ancestry were extracted for data analysis. The study included 12,882 IBD participants and 21,770 healthy controls. Its subgroup UC included 6986 patients and 20464 controls; the study included 5956 CD patients and 14927 healthy controls (22). On established histological, endoscopic, and imaging standards, IBD and its subtypes are diagnosed. The FinnGen database collected genetic risk variants for hidradenitis suppurativa (HS) (23). The database comprises 409 cases diagnosed with HS and 211,139 controls without HS, which were analyzed according to the criteria for ICD10 (International Classification of Diseases).

### SNP selection

We selected the most significant and independent SNP (*p*<5×10^-8^, linkage disequilibrium (LD) r^2^<0.001) as the primary analysis. For the exposure analysis of IBD and its two major subtypes, 61 SNPs were associated with IBD, 39 SNPs were associated with UC, and 53 SNPs were selected for association with CD. At a threshold of *p*<5x10^-8^, less than 10 SNPs were screened for HS and therefore failed to meet the minimum criteria for MR analysis (24). Under a more relaxed threshold of *p*<5x10^-6^ *(* 25), 14 SNPs associated with HS were screened. Due to the lower significance criterion, The risk of light instrument bias was assessed using the F statistic(F<10). Selected SNPs were additionally matched to the human genotype-phenotype association database (phenoscanner) using a threshold of *p*<5x10^-6^ to prevent potential relationships between SNPs and outcome confounders (26, 27). Prior to performing the MR analysis, the exposure and outcome data were harmonized by aligning the SNPs on the same effect allele. Details regarding the IVS are presented in **Supplementary Tables 1–4**.

### MR estimates

The MR analysis was run in two different orientations. The first one genetically predicts the causal effect of IBD on HS, while the second one genetically predicts the inverse outcome of HS on IBD. Standard MR analysis employs the inverse variance weighted model (IVW), where all genetic variances are taken to be reliable instruments. Thus, the IVW estimate can be obtained by weighted least-square regression with no intercept, in which the slope is the causal estimate (28). The weighted median estimation model and the MR-Egger regression model were also created to estimate the causal linkages under various circumstances. Based on the weighted median estimation approach, when half of the IVs are valid, the unbiased impact of all available SNPs is estimated (29). In MR-Egger regression models, the slope and intercept are adjusted for pleiotropy, so the estimates are relatively robust regardless of the validity of the IV (30).

The results’ reliability and stability were evaluated using heterogeneity, pleiotropy, and sensitivity analyses. Cochrane’s Q statistic assessed the IVs’ heterogeneity; *p*<0.05 values denote significant heterogeneity, in which case a random effects model was applied to the remaining study. If not, a fixed effects model is applied (31). To assess the consistency of the MR results, IVs were eliminated one by one (29). The MR-PRESSO test is a widely used method to test horizontal multi-effectiveness. The MR-PRESSO outlier test compares the distance from a single SNP to the fitted line, and the larger the distance, the more it indicates that this SNP may be an outlier that we need to remove (32). Using the intercepts acquired from the study of the MR-Egger regression model, one may determine the direction of multiplicity and make corrections. However, the MR-Egger regression mode only serves as a complement to the IVW approach because it produces wider confidence intervals (CI). As a result, MR-Egger regression models were applied in instances of significant pleiotropy, and MR-PRESSO models were utilized to identify outliers. Otherwise, the IVW outcomes took precedence.

To avoid false positive results in two-way multiple tests, Bonferroni correction was performed. After Bonferroni correction, the bilateral *p* value <0.017 was statistically significant, and *p* values ≥ 0.017 and<0.05 suggested significance. All analyses were performed by the two-sample MR package (version 0.5.6) and the MR-PRESSO package (version 1.0) of the R program (version 4.1.3).

## Results

### Causal effects of IBD or its main subtypes on hidradenitis suppurativa

After strict exclusion criteria, there was no weak instrumental variable bias, as evidenced by the F-statistics being larger than 10 for the three IVs of IBD, UC, and CD. In the MR-PRESSO tests, no outlier SNPs were discovered.

**Figure 2:**
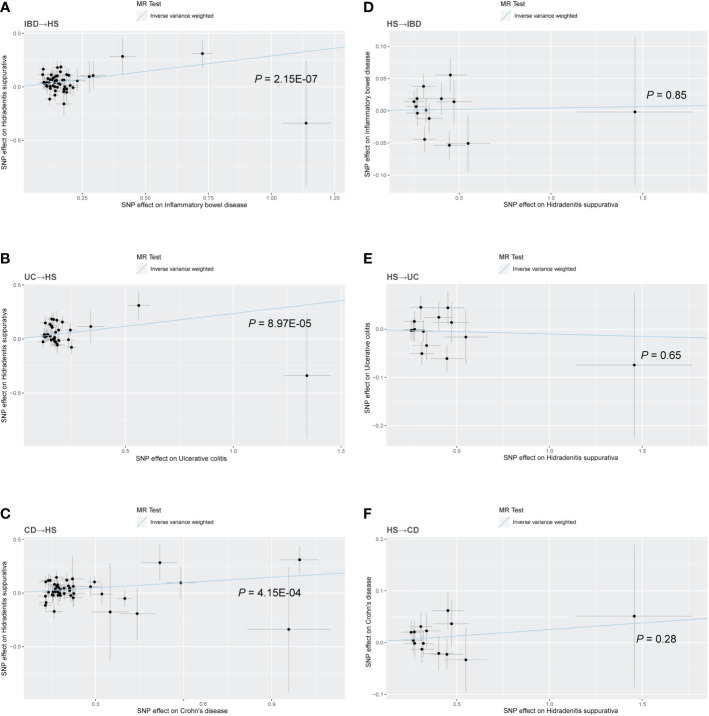
Scatter plot of the MR analysis. The slope of each line corresponds to the MR effect estimated by the IVW model. **(A)** IBD on HS; **(B)** UC on HS; **(C)** CD on HS; **(D)** HS on IBD; **(E)** HS on UC; **(F)** HS on CD.

There was no heterogeneity in Cochran’s Q test for IBD and its subtypes (*p* > 0.05). Therefore, we use a fixed effect model based on the IVW method. We discovered a clear causal link between IBD and HS (IVW OR = 1.34, 95% CI = 1.20-1.49, *p* = 2.15E-07, **Figure 2A**). Weighted median results were consistent with IVW results (Weighted median OR = 1.42, 95% CI = 1.20-1.68, *p* = 5.81E-05). According to the MR-Egger method, IBD does not cause HS (MR Egger OR = 1.26, 95% CI = 0.91-1.76, *p* = 0.17). The IVW estimates were consistent with the Weighted median estimates. The pleiotropy test did not show statistical significance (*p* > 0.05 for the MR Egger intercept), which suggests that the IVW estimates may be more reliable in assessing causal effects than the MR Egger regression estimates.

In addition, a significant correlation was found between UC and HS (IVW OR = 1.27, 95% CI = 1.13-1.43, *p* = 8.97E-04, **Figure 2B**; Weighted median OR = 1.24, 95% CI = 1.10-1.46, *p* = 0.02). OR did not differ from IVW and Weighted median, although MR Egger did not find a statistically significant difference (MR Egger OR = 1.12, 95% CI = 0.69-1.80, *p* = 0.65). The three methods of IVW and Weighted median and MR Egger showed statistically significant causal effects of CD on HS (IVW OR = 1.18, 95% CI = 1.08-1.29, *p* = 4.15E-04, **Figure 2C**; Weighted median OR = 1.22, 95% CI = 1.07-1.40, *p* = 0.0036; MR Egger OR = 1.27, 95% CI = 1.01-1.60, *p* = 0.04). The results show a higher confidence level.

**Figure 3:**
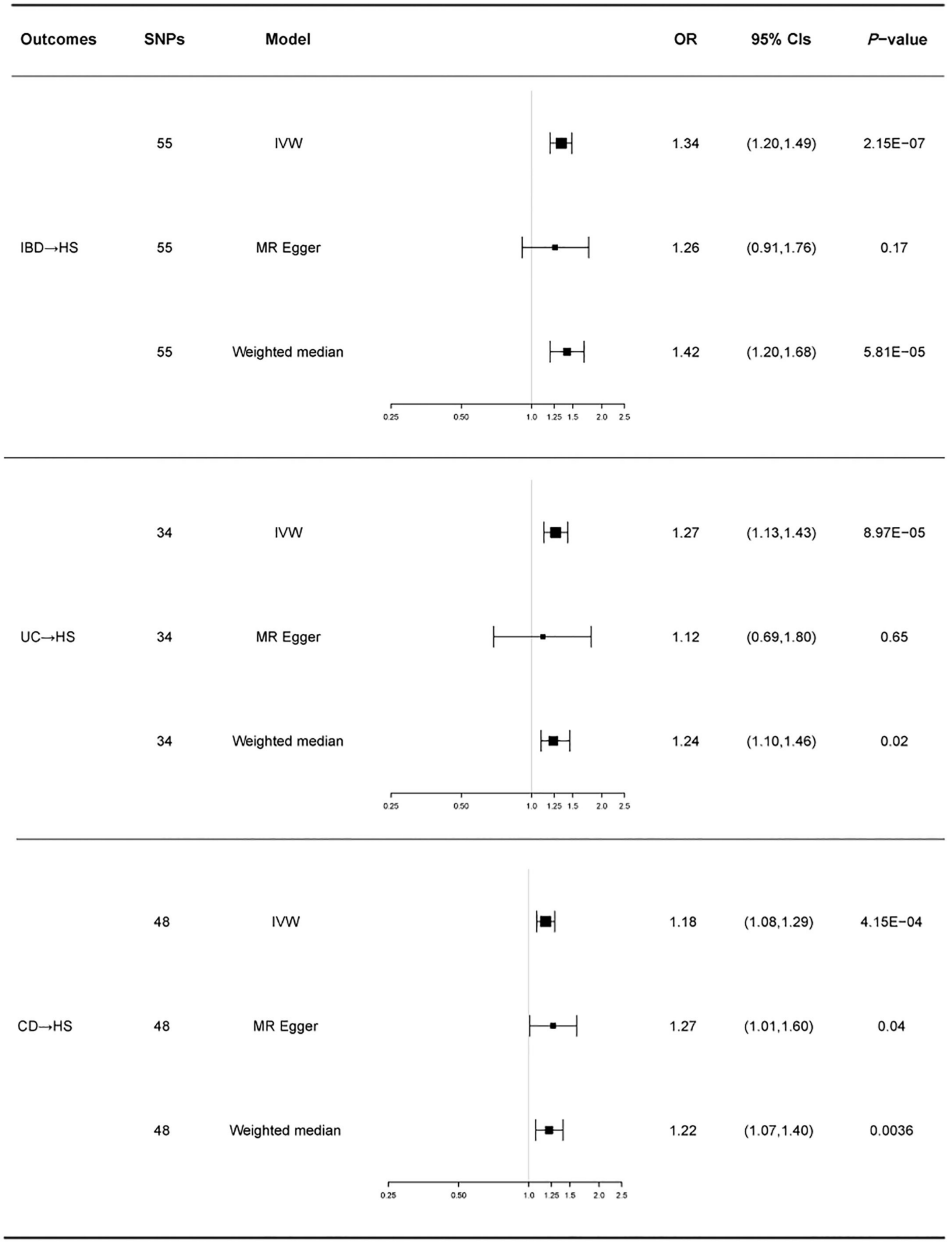
For the effects of inflammatory bowel disease, ulcerative colitis, and Crohn’s disease on hidradenitis suppurativa, causal estimates are given as odds ratio (OR) and 95% confidence interval (CI). IBD, inflammatory bowel disease; UC, ulcerative colitis; CD, Crohn’s disease; HS, hidradenitis suppurativa.

**Figure 3** shows a forest plot of the causal relationship between genetically predicted IBD and its subtypes and HS. Details of the sensitivity analysis are presented in **Table 1**, the leave-one-out plot is depicted in **Supplementary Figure 1**, and the funnel plot is depicted in **Supplementary Figure 2**.

**Table 1:** Sensitivity analyses of MR.

Exposure	Outcome	SNPs	Heterogeneity test		MR Egger pleiotropy test		MR-PRESSO global outlier test
			Q	*P*-value	Intercept	*P*-value	Outlier
IBD	HS	55	60.4678	0.25	0.0093	0.72	None
UC	HS	34	46.1268	0.06	0.0234	0.59	None
CD	HS	48	48.6769	0.41	-0.0178	0.46	None
HS	IBD	14	22.8733	0.04	0.0205	0.49	None
HS	UC	14	16.4250	0.23	0.0155	0.63	None
HS	CD	14	7.34106	0.88	0.0096	0.74	None

MR, Mendelian randomization analysis; SNPs, Number of single nucleotide polymorphism. IBD, inflammatory bowel disease; UC, ulcerative colitis; CD, Crohn’s disease; HS, hidradenitis suppurativa.

### Causal effects of hidradenitis suppurativa on IBD or its main subtypes

HS was used as the exposure factor to demonstrate reverse causality to indicate IBD’s outcome. The F-statistic for all IVs was>10, indicating no bias for weak instrumental variables. All MR Egger regressions delivered negative findings (*p* for MR-Egger pleiotropy test > 0.05), indicating the absence of directional pleiotropy effect bias. MR-PRESSO tests did not reveal any outlier SNPs.

Similarly, we used fixed-effects models for the UC and CD groups and random-effects models for the IBD group. Using the IVW approach, HS showed no causative influence on IBD (IVW OR = 1.00, 95% CI = 0.96-1.05, p = 0.85, **Figure 2D**). Additionally, we were unable to uncover any proof linking HS with UC (IVW OR = 0.99, 95% CI = 0.95-1.03, p = 0.65, **Figure 2E**). HS and CD are also not related (IVW OR = 1.03, 95% CI = 0.98-1.07, p = 0.28, **Figure 2F**). Other MR methods suggest the same results.

**Figure 4:**
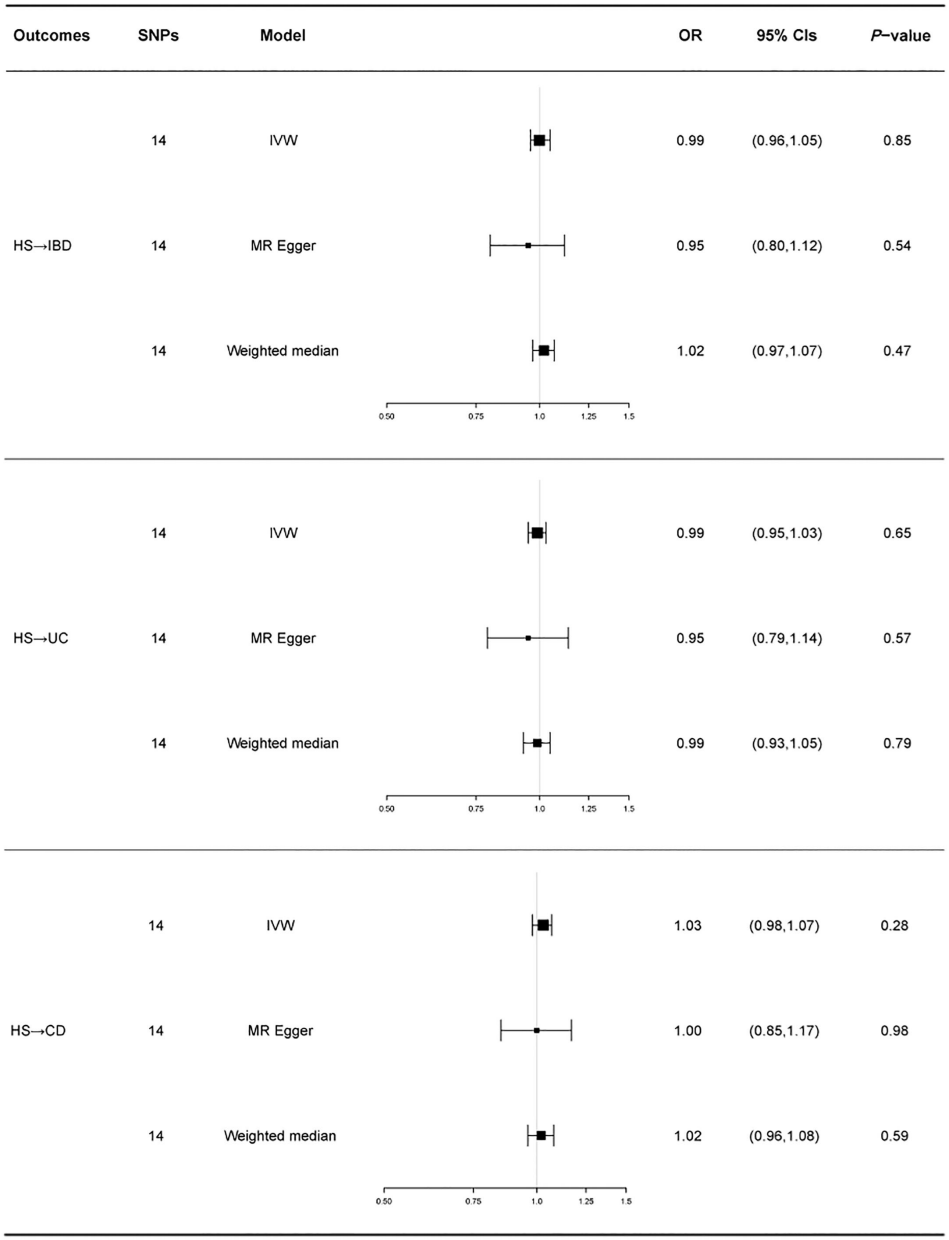
For the effect of hidradenitis suppurativa on inflammatory bowel disease, ulcerative colitis and Crohn’s disease, causal estimates are given as odds ratio (OR) and 95% confidence interval (CI). IBD, inflammatory bowel disease; UC, ulcerative colitis; CD, Crohn’s disease; HS, hidradenitis suppurativa.

A forest plot of the causality of gene prediction HS for IBD and its subtypes is shown in **Figure 4**. Details of the sensitivity analysis are shown in **Table 1**, the leave-one-out plot is exhibited in **Supplementary Figure 3**, and the funnel plot is exhibited in **Supplementary Figure 4**.

## Discussion

With the help of pooled GWAS data, the current study thoroughly investigated the causal link between IBD and HS. To the extent of our knowledge, this study is the first to examine the reciprocal causal link between IBD and HS using MR techniques. According to this bidirectional MR study, IBD and its subtypes (UC and CD) are causally related to HS. Previous studies did not suggest any association between UC and HS (10, 18), which seemed inconsistent with the results of our analysis. It is important to note that IBD and HS result from genetic and environmental factors. The environment may alter the expression and function of different genes, and environmental factors also contribute to the emergence of these disorders. A case-control study by Parul Tandon et al (33). showed that smoking, obesity, and alcohol consumption are important ecological factors in the pathogenesis of IBD and HS. Gene-gene, gene-environment, and environment-environment interactions cause discrepancies between the findings of observational studies and the results of MR analyses. In addition, in diseases where genetic susceptibility is not the only causal factor, the MR-Egger method results in a broader range of confidence intervals, leading to a negative causal relationship between IBD and HS.

However, there was no evidence of a causal link between IBD and its subtypes when HS was utilized as an exposure factor. Two meta-analyses have examined the medical literature’s relationship between HS and IBD. Eight observational studies in a meta-analysis (7 case-control or cross-sectional studies and 1 cohort study) (34), including 93601 study participants. IBD and HS were found to be significantly correlated in two case-control studies (OR, 2.16 [95% CI, 1.40-3.34] and 10.00 [95% CI, 1.94-51.50]). In a cohort study, people with HS had a higher risk of IBD (HR,5.6; 95% confidence interval not reported; *p* < 0.002). Studies in case-control and cross-sectional design revealed a strong correlation between HS and ulcerative colitis (pooled OR, 1.51; 95% CI, 1.25-1.82) and Crohn’s disease (pooled OR, 2.12; 95% CI 1.46-3.08). Furthermore, Kevin Phan et al. (35) Included 1782 subjects reporting an association between IBD and HS. After combining adjusted effect sizes, there was a significant association between IBD and HS (OR 2.12; 95% CI 1.62-2.77). Subgroup analysis revealed significant associations between HS and UC (OR 1.56; 95% CI 1.26-1.94) and CD (OR 2.25; 95% CI 1.52-3.32). These reported estimates differ from our findings in some ways. We believe the discrepancies may be due to differences in analytical methods. Observational studies may be influenced by inevitable clinical confounders that may impact exposure and results and limit the capacity to draw precise conclusions about the causes of observed outcomes. Therefore, even if an observational study finds a significant connection, this does not imply a direct causal link. By introducing genetic instrumental variables, Mendelian randomization approaches can avoid the impact of these confounding factors, producing a rather accurate causality determination.

Genetic factors play an important role in the development and progression of disease. The concordance rate of UC can reach 18.2% between monozygotic twins and up to 58.3% in CD (36). Twin studies from the Netherlands and Denmark also reported a consistent prevalence of HS in monozygotic twins of 28%, with biometric models suggesting heritability of 77%-80% (37, 38). Common genetic susceptibility loci have been identified between IBD and HS. Specific genes, such as ELOVL7 (OMIM 614451) and SULT1E1 (OMIM 600043), and SULT1B1 (OMIM 608436), are related to IBD and HS (39). Gene pathway enrichment analysis of the SNPs screened in this MR study revealed that they were associated with T cell activation, transcription factor activation, tumor necrosis factor (TNF), and interleukin release. Studies have shown that IBD and HS are diseases of immune dysregulation. Abnormal expression of cytokines such as TNF and interleukin 1, interleukin 6, interleukin 17, and interleukin 23 are involved in the pathogenesis of both IBD and HS (40–42). Janus kinase (JAK)-signal transducer and activator of transcription (STAT) DNA binding mediates the pathogenesis of IBD (43). Tofacitinib, a pan-JAK inhibitor, had distinct efficacy in UC and CD patients, indicating that the JAK/STAT pathway was activated differently in each group of patients (44, 45). STAT1 signaling in T cells was elevated in CD but not in UC, and STAT1 mRNA was elevated in HS patients (46, 47). This shows that STAT1 may function as a mediator between UC, CD, and HS. In addition, alterations in the microbiota may contribute to systemic immune damage. This close interaction between gut flora, cytokines, and skin lesions has been proposed as a theory of the gut-skin axis. However, the current study’s fin does not support the notion that IBD and HS interact in a bidirectional manner, indicating that this kind of communication along the gut-skin axis is not particularly reversible. IBD has a more direct effect on skin function, whereas HS cannot cause IBD *via* a gut-skin axis pathway.

Our study has three benefits. First, the MR design and data from a sizable GWAS were used to reduce the impact of unmeasured covariates and reverse causality. Secondly, we performed a stratification study and found that both IBD and its subtypes were associated with HS, increasing the stability of the results. Third, the robustness of MR data is provided through sensitivity analysis and multipotency testing utilizing several MR methodologies. This study also has limitations. The majority of the study sample’s participants were of European descent. The study’s outcomes may not be as generalizable to different populations as a result. In contrast, the prevalence and incidence of IBD are significantly higher in Europeans than in Asians (48). Other populations should be studied to determine the causal relationship between IBD and HS. Furthermore, due to the lack of IVs, a lower p threshold (p<5x10^-6^) was selected for the HS study. Also, in the FinnGen database, HS was confirmed in only 409 cases. Despite the fact that our F-statistical test revealed no significant risk of bias, this negative outcome needs to be evaluated with care. Last but not least, to exclude any confounding variables and related horizontal pleiotropy, we set all chosen SNPs into the phenoscanner database. However, this precaution does not eliminate horizontal pleiotropy. The exact biological function of many SNPs remains unknown. Future studies will overcome these issues when higher-quality GWAS research is made accessible.

## Conclusions

The present study provides genetic evidence that IBD and its subtypes have a causal effect on HS, while HS does not affect IBD. Gut-skin axis interactions may help to understand this association. Nonetheless, additional research is required to clarify the pathophysiology of the causative link between IBD and HS.

## Data availability statement

The datasets presented in this study can be found in online repositories. The names of the repository/repositories and accession number(s) can be found in the article/**Supplementary Material**.

## Author contributions

BB contributed to data collection, analysis, and writing of the manuscript. CZ contributed to data analysis. JS contributed to data collection, and CL contributed to the study design. All authors contributed to the article and approved the submitted version.

## Supplementary material

Supplementary Figure 1 Leave one out of sensitivity tests of MR analyses of inflammatory bowel disease, ulcerative colitis, and Crohn's disease on hidradenitis suppurativa. Calculate the MR results of the remaining IVs after removing the IVs one by one. **(A)** IBD on HS; **(B)** UC on HS; **(C)** CD on HS. IBD, inflammatory bowel disease; UC, ulcerative colitis; CD, Crohn’s disease; HS, hidradenitis suppurativa.

Supplementary Figure 2 Funnel plot of the MR analysis. **(A)** IBD on HS; **(B)** UC on HS; **(C)** CD on HS.

Supplementary Figure 3 Leave one out of sensitivity tests of MR analyses of hidradenitis suppurativa on inflammatory bowel disease, ulcerative colitis, and Crohn's disease. Calculate the MR results of the remaining IVs after removing the IVs one by one. **(A)** HS on IBD; **(B)** HS on UC; **(C)** HS on CD. IBD, inflammatory bowel disease; UC, ulcerative colitis; CD, Crohn’s disease; HS, hidradenitis suppurativa.

Supplementary Figure 4 Funnel plot of the MR analysis. **(A)** HS on IBD; **(B)** HS on UC; **(C)** HS on CD.
