# Supplementary figures and images for: Causal association between inflammatory bowel disease and hidradenitis suppurativa: A two-sample bidirectional Mendelian randomization study
Source: Front Immunol. 2023 Jan 26;14:1071616. doi: 10.3389/fimmu.2023.1071616 (PMC9909343; doi:10.3389/fimmu.2023.1071616)

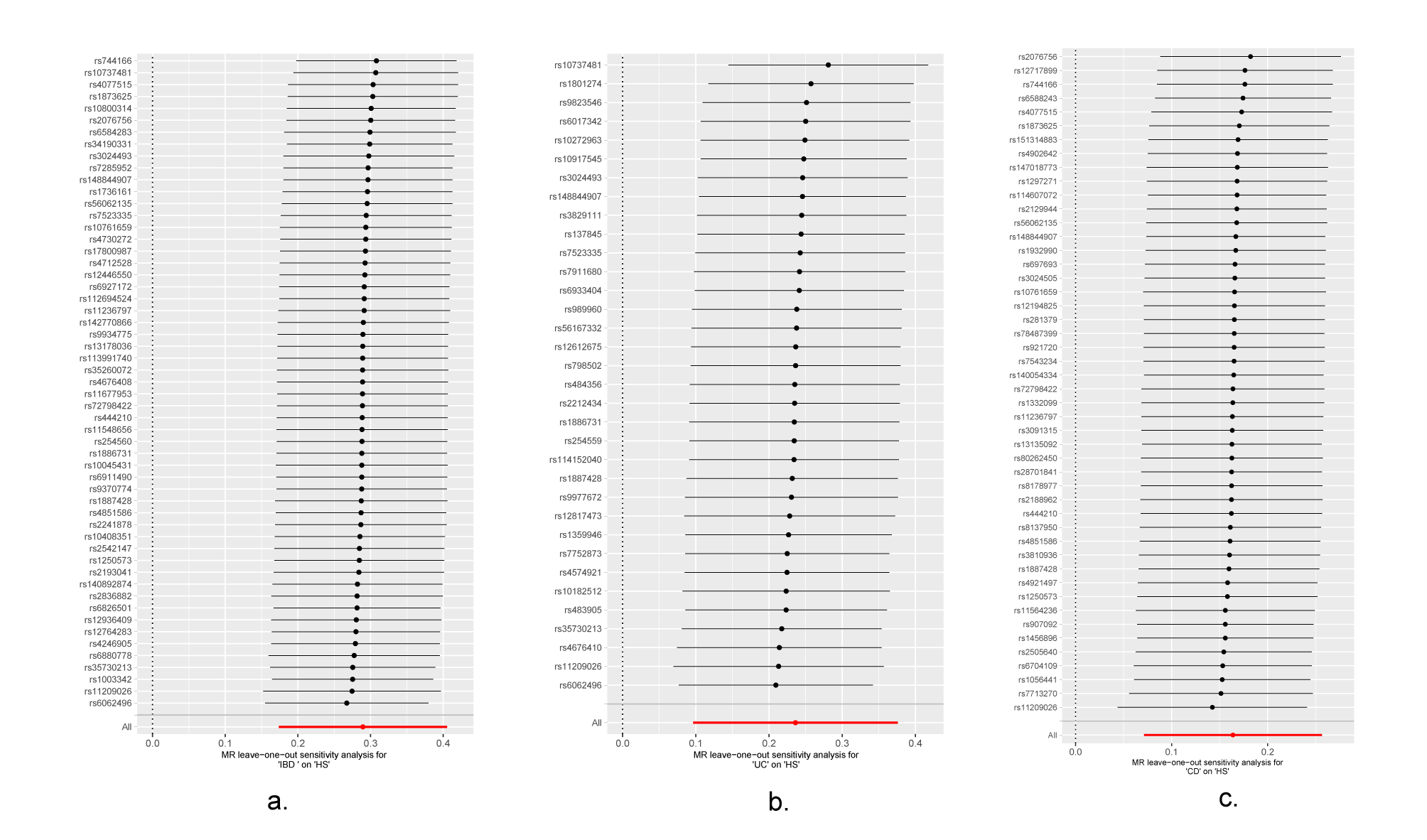

Supplement: Supplementary Figure 1 — Leave one out of sensitivity tests of MR analyses of inflammatory bowel disease, ulcerative colitis, and Crohn's disease on hidradenitis suppurativa. Calculate the MR results of the remaining IVs after removing the IVs one by one. (A) IBD on HS; (B) UC on HS; (C) CD on HS. IBD, inflammatory bowel disease; UC, ulcerative colitis; CD, Crohn’s disease; HS, hidradenitis suppurativa. [file DataSheet_1.zip › Figure/Supplementary Figures 1.tif]

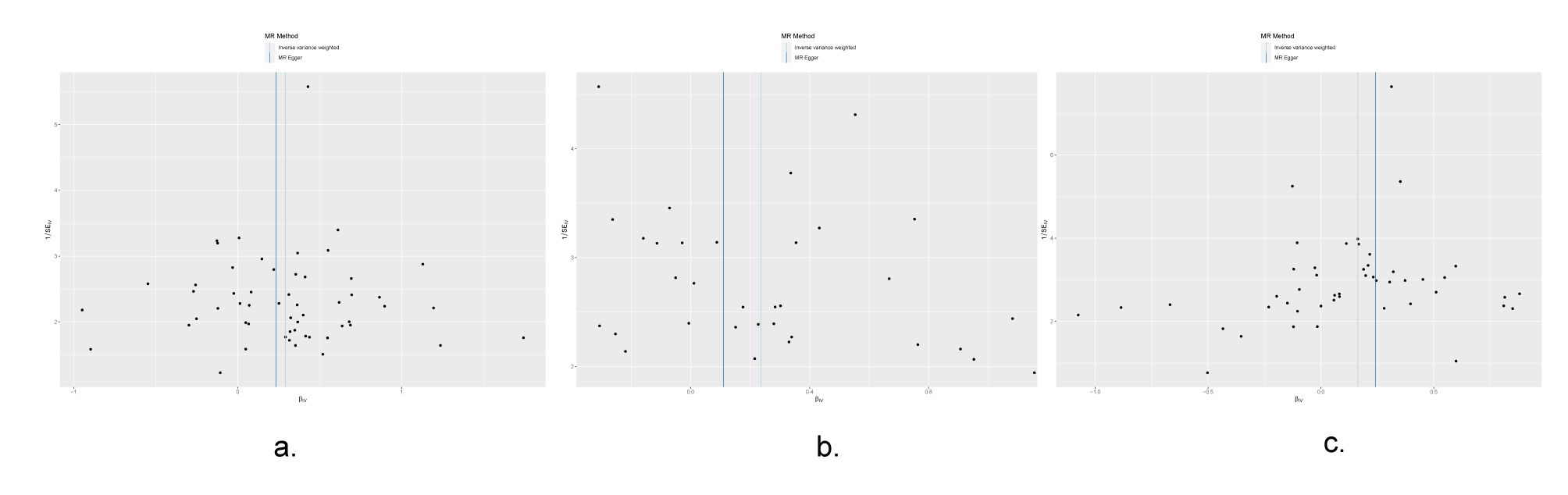

Supplement: Supplementary Figure 1 — Leave one out of sensitivity tests of MR analyses of inflammatory bowel disease, ulcerative colitis, and Crohn's disease on hidradenitis suppurativa. Calculate the MR results of the remaining IVs after removing the IVs one by one. (A) IBD on HS; (B) UC on HS; (C) CD on HS. IBD, inflammatory bowel disease; UC, ulcerative colitis; CD, Crohn’s disease; HS, hidradenitis suppurativa. [file DataSheet_1.zip › Figure/Supplementary Figures 2.tif]

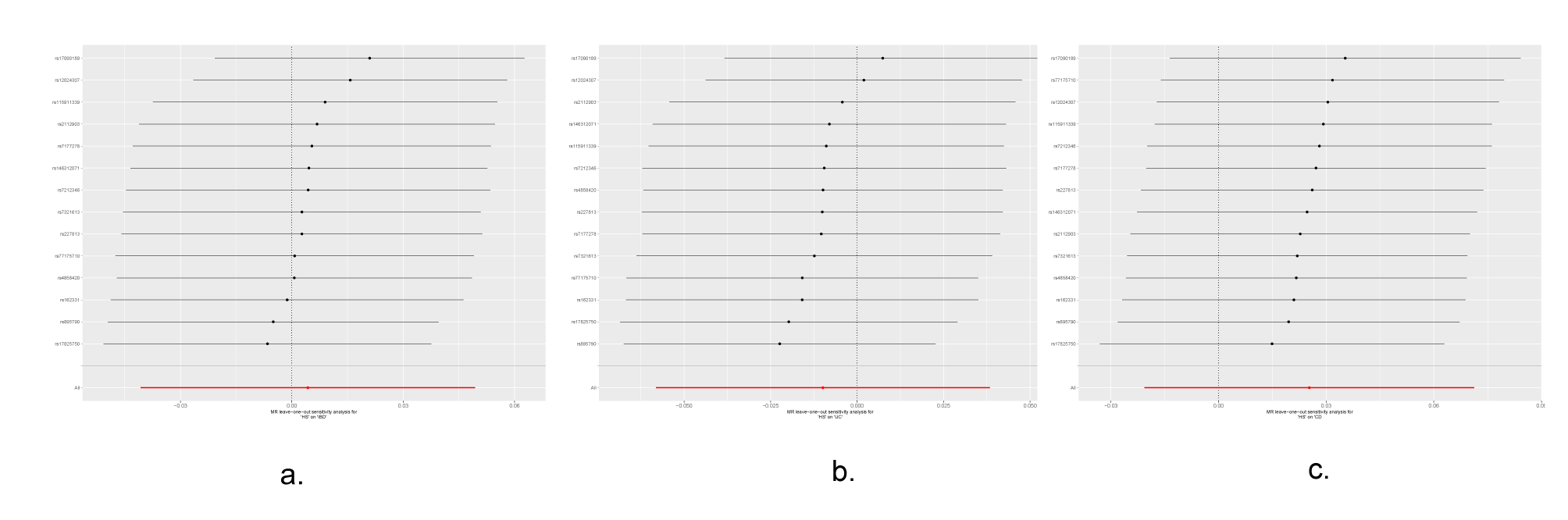

Supplement: Supplementary Figure 1 — Leave one out of sensitivity tests of MR analyses of inflammatory bowel disease, ulcerative colitis, and Crohn's disease on hidradenitis suppurativa. Calculate the MR results of the remaining IVs after removing the IVs one by one. (A) IBD on HS; (B) UC on HS; (C) CD on HS. IBD, inflammatory bowel disease; UC, ulcerative colitis; CD, Crohn’s disease; HS, hidradenitis suppurativa. [file DataSheet_1.zip › Figure/Supplementary Figures 3.tif]

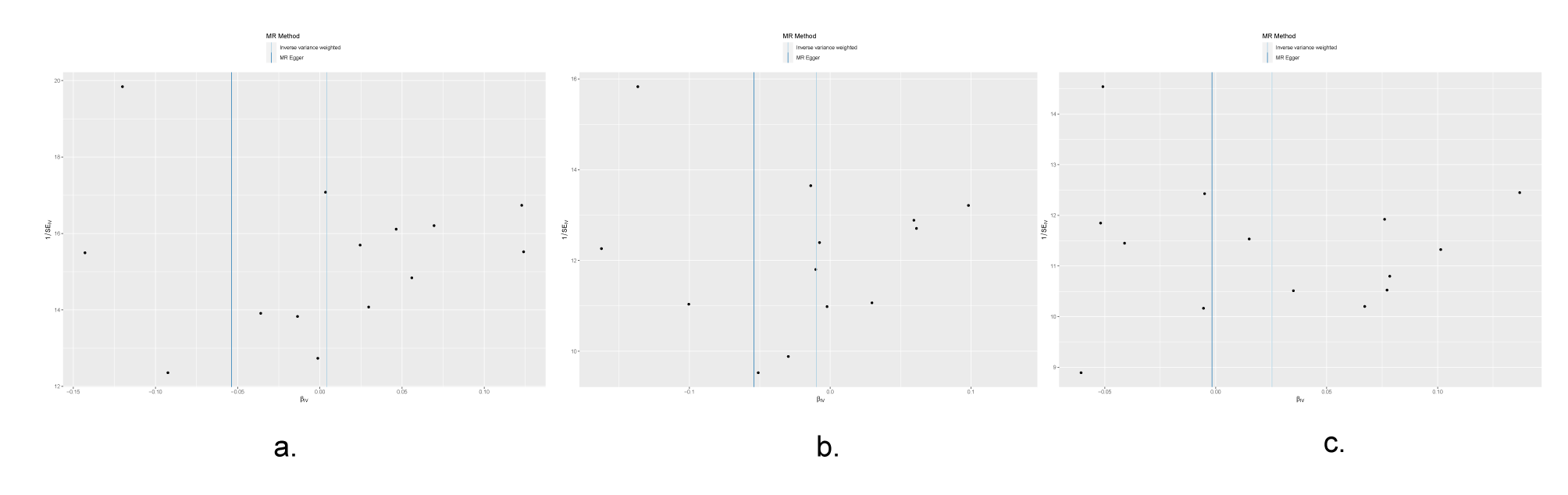

Supplement: Supplementary Figure 1 — Leave one out of sensitivity tests of MR analyses of inflammatory bowel disease, ulcerative colitis, and Crohn's disease on hidradenitis suppurativa. Calculate the MR results of the remaining IVs after removing the IVs one by one. (A) IBD on HS; (B) UC on HS; (C) CD on HS. IBD, inflammatory bowel disease; UC, ulcerative colitis; CD, Crohn’s disease; HS, hidradenitis suppurativa. [file DataSheet_1.zip › Figure/Supplementary Figures 4.tif]
